# A systematic review and Meta-analysis of urinary extracellular vesicles proteome in diabetic nephropathy

**DOI:** 10.3389/fendo.2022.866252

**Published:** 2022-08-11

**Authors:** Xiaonan Ding, Xiaochen Wang, Junxia Du, Qiuxia Han, Dong Zhang, Hanyu Zhu

**Affiliations:** ^1^ Medical School of Chinese People’s Liberation Army (PLA), Beijing, China; ^2^ Department of Nephrology, The First Medical Center, Chinese People’s Liberation Army (PLA) General Hospital, Chinese People’s Liberation Army (PLA) Institute of Nephrology, State Key Laboratory of Kidney Diseases, National Clinical Research Center for Kidney Diseases, Beijing, China

**Keywords:** diabetic nephropathy, urinary extracellular vesicles, exosomes, microvesicles, proteome

## Abstract

Diabetic nephropathy (DN) is a major microvascular complication of both type 1 and type 2 diabetes mellitus and is the most frequent cause of end-stage renal disease with an increasing prevalence. Presently there is no non-invasive method for differential diagnosis, and an efficient target therapy is lacking. Extracellular vesicles (EV), including exosomes, microvesicles, and apoptotic bodies, are present in various body fluids such as blood, cerebrospinal fluid, and urine. Proteins in EV are speculated to be involved in various processes of disease and reflect the original cells’ physiological states and pathological conditions. This systematic review is based on urinary extracellular vesicles studies, which enrolled patients with DN and investigated the proteins in urinary EV. We systematically reviewed articles from the PubMed, Embase, Web of Science databases, and China National Knowledge Infrastructure (CNKI) database until January 4, 2022. The article quality was appraised according to the Newcastle-Ottawa Quality Assessment Scale (NOS). The methodology of samples, isolation and purification techniques of urinary EV, and characterization methods are summarized. Molecular functions, biological processes, and pathways were enriched in all retrievable urinary EV proteins. Protein-protein interaction analysis (PPI) revealed pathways of potential biomarkers. A total of 539 articles were retrieved, and 13 eligible records were enrolled in this systematic review and meta-analysis. And two studies performed mass spectrometry to obtain the proteome profile. Two of them enrolled only T1DM patients, two studies enrolled both patients with T1DM and T2DM, and other the nine studies focused on T2DM patients. In total 988 participants were enrolled, and DN was diagnosed according to UACR, UAER, or decreased GFR. Totally 579 urinary EV proteins were detected and 28 of them showed a potential value to be biomarkers. The results of bioinformatics analysis revealed that urinary EV may participate in DN through various pathways such as angiogenesis, biogenesis of EV, renin-angiotensin system, fluid shear stress and atherosclerosis, collagen degradation, and immune system. Besides that, it is necessary to report results compliant with the guideline of ISEV, in orderto assure repeatability and help for further studies. This systematic review concordance with previous studies and the results of meta-analysis may help to value the methodology details when urinary EV proteins were reported, and also help to deepen the understanding of urinary EV proteins in DN.

## Introduction

Diabetic nephropathy (DN) is a serious microvascular complication of type 1 diabetes mellitus (T1DM) and type 2 diabetes mellitus (T2DM). It is the one of most prevalent causes of chronic kidney disease (CKD) and accounts for 25% to 40% of patients with end-stage renal disease (ESRD) in Western countries (1, 2). DN is generally clinically diagnosed based on the presence of albuminuria and/or a reduced estimated glomerular filtration rate (eGFR) without signs or symptoms of other primary causes of renal damage (3). However, the decline in eGFR is not always in concordance with increased albuminuria, and the converse progression from microalbuminuria to normoalbuminuria can also be observed (4). Differential and clear diagnoses are usually recognized by renal biopsy; specifically, it is necessary that non-invasive biomarkers surrogate albuminuria to diagnose and prognose DN (5). The interventions for DN include nutrition management, glycemic and hypertension control, the use of direct renal effects of glucose-lowering medications, and mineralocorticoid receptor, antagonists. Therefore, there is an absence of a targeted medicine for DN therapy (2). Despite extensive studies, it is also important to further expand the edge of the biomarkers of responsiveness to regimens.

To date, numerous studies explore the frontiers of DN, and extracellular vesicles (EV) contain large information as a reliable resource to further comprehensive disease mechanisms. EV is a heterogeneous group of spherical bilayered proteolipid structures originating from basically all cell types, harboring various biological cargo such as proteins, lipids, and unfunctional nucleus, reflecting the original cells’ physiological states and pathological conditions. EV is composed of exosomes (50–150 nm in diameter, originating from the endosomal system and plasma membrane fusion for release), microvesicles (50–1000 nm in diameter, budding from the plasma membrane), and apoptotic bodies(800–5000 nm in diameter, shed from dying cells). And EV can be detected in a variety of body fluids such as blood, urine, cerebrospinal fluid, etc. and the key advantage of urine over other EV-containing fluids is it is produced directly by the kidneys and can be collected in large quantities noninvasively (6). And in the urinary EV can be identified proteins not found in the whole urine, because the concentration of these proteins in the whole urine is low or they are located inside of urinary EV (7). Therefore, using urinary EV to look for noninvasive biomarkers may be more advantageous than whole urine, and may provide new perspectives on the pathological mechanisms of DN. Urinary EV come from various sources, such as kidneys, bladder, and immune cells that reside in the genitourinary tract, besides that, some EV enter the urine from the circulatory system (8). In the physiological state, only a diameter of less than 6 nm in the circulation can enter the urine through the glomerular filtration barrier, while the average diameter of exosomes is about 40-150 nm; However, in pathological conditions, podocyte damage and basement membrane are injured, providing conditions for circulating EV to enter the urine (9). Thus, the diversity of urinary EV sources determines that it can comprehensively reflect the pathophysiology of the kidney (10).

EV is involved in various biochemical processes and is speculated to remove excess and/or unnecessary constituents from cells to maintain homeostasis, regulate intercellular communication, and participate in immune responses and infection (8, 11). And almost all kinds of renal cells can release EV, such as podocytes, glomerular endothelial cells, tubular cells, and so on. When cells are under the stress condition of a diabetic internal environment, the amounts and contents of EV are corresponding changed involved with cross-talk the density and size also can reflect the deteriorated kidney function (12, 13).

**Figure 1:**
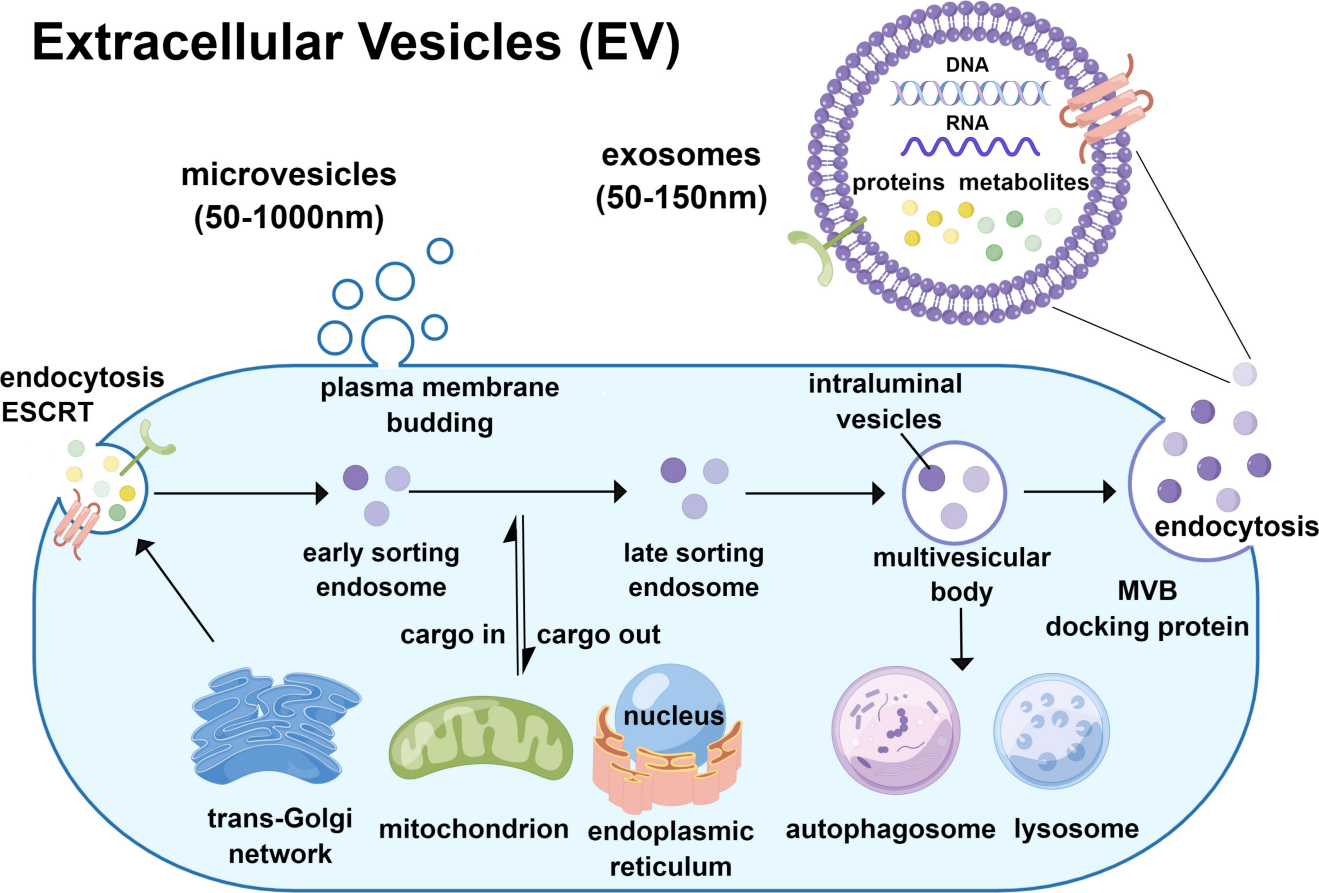
Biogenesis of exosomes and microvesicles.

Nowadays, the terminology of EV generally covers exosomes and microvesicles. Although exosome and microvesicle are similar in biophysics, as the subpopulation of EV, they differ in biogenesis (10). Exosome biogenesis is generated by the plasma membrane invagination or, in some cases, from the trans-Golgi network (TGN). In this process, the components such as proteins, lipids, and nucleic acids are selectively encased under the guidance of the endosomal sorting complex required for the transport protein family (ESCRT) to form early sorting endosomes (ESEs), which can fuse with each other and can also perform cargo-in and cargo-out with organelles such as the endoplasmic reticulum, TGN, and mitochondria. Some ESEs then develop as late sorting endosomes (LSEs) (14). Invagination of LSEs leads to the synthesis of intraluminal vesicles (ILVs), which are further clustered and wrapped to form multivesicular bodies (MVBs). After that, the alternative part of it ultimately fuses with autophagosomes or lysosomes to undergo degradation, and another part takes intracellular trafficking to the plasma membrane with the help of MVB-docking protein, releasing the containing ILVs to the extracellular milieu by exocytosis as an exosome (15–17). Microvesicles are also known as microparticles or ectosomes, directly generated by outward budding and rely on the contraction of actomyosin and phosphorylation of myosin (18). Many studies have investigated the mechanisms of microvesicle biogenesis and release and have shown that the ESCRT machinery also plays an important role in exosome biogenesis (19, 20). The release of microvesicles is regulated by calcium (Ca2^+^) concentration and the accumulation of protein-degrading enzymes (18). The biogenesis process were shown in **Figure 1**. Considering the different biogenesis of exosomes and microvesicles, which probably determines their composition and function, it is necessary to identify the subgroup of EV for further study.

Therefore the characterization of EV shows an important role. According to the International Society for Extracellular Vesicles guidelines, EV should be characterized based on morphology, size distribution and counts, and EV content, such as proteins. The morphology can be examined using transmission electron microscopy (TEM) or atomic force microscopy (AFM), and the particle diameter distribution and concentration can be directly defined using nanoparticle tracking analysis (NTA). Biomarkers used to characterize urinary EV are usually identified by western blotting(WB) (21). However, some widely used biomarkers overlap in exosomes and microvesicles, such as CD9, CD63, CD81, CD82, ALIX, and TSG101, which often cannot represent the uniqueness of specific subgroups of urinary EV (22, 23). Several proteins had been recommended to evaluate exosomes based on the procession of biogenesis which involved various organelles, such as the nucleus (HIST1H and LMNA), mitochondria (IMMT, CYC1, TOMM20), endoplasmic reticulum, and Golgi apparatus (CANX, HSP90B1, HSPA5, and GOLGA2), autophagosomes and cytoskeleton (ATG9A, ACTN1/4, and KRT18) (21).

So far, studies about the standard of methodology in urinary EV are still limited, especially regarding the type of samples and protocols for isolation of urinary EV. The difference in methodology might contribute to the heterogeneity among studies, which obstacle the reliability and reproducibility of published EV results (8). There is not an approach applicable to all kinds of studies, in addition, the procedure for obtaining EV is always accompanied by a dilemma not only in purification and yield but also in time consumption, labor intensity, and expensive equipment (10, 22). When it comes to urine that Tamm-Horsfall protein (THP) and albumin were commonly co-precipitated with EV to decrease the purification and yield. The choice of an isolation method is dependent on the type of urine sample (non-proteinuria or proteinuria) and the type of downstream analyses used (10). Considering the particularity of urine, the following separation and concentration techniques were the most commonly used.

- **Differential ultracentrifugation:** Ultracentrifugation is the broadest method for EV isolation with variant protocols, combined with other purification techniques such as density gradients, filtration, and immunoprecipitation. It generally commences with low-speed centrifugation at 300–400 × g to remove cellular debris and apoptotic bodies and intermediate speed spin at 1,000–2,000 × g to precipitate large EVs such as microvesicles and polymerized THP (24). Relatively small EVs such as exosomes with low density remained in the supernatant and precipitated further by high-speed spin (100,000–200,000 × g).
- **Ultracentrifugation combined with DTT/CHAPS:** Although polymerized THP is expected to be removed in sedimentation during intermediate speed spin, substantial EVs are entrapped by the polymerized THP because of the construction of filaments and matrices (25). Because of this highly abundant protein co-precipitation with EV, the yield can be decreased. Therefore, additional methods, such as dithiothreitol (DTT) are used to eliminate the disulfide bonds of polymerized THP, and alkaline buffers were added to release the entrapped EV (10).
- **Ultracentrifugation combined with density gradient:** Based on the different densities of exosomes, microvesicles, and debris that EV could travel to the equilibrium density during the ultracentrifugation. Therefore, the gradient separates the subpopulations of EV which can be used to further purify, and sucrose was the most commonly used to create the density gradient (26).
- **Size exclusion chromatography (SEC):** SEC technique separates EV based on the different sizes of EV and debris, the components or vesicles smaller than the gap of the porous matrix can be fractionated and relatively larger contaminants can be removed. But this method fails to concentrate EV, therefore, it is commonly performed combined with ultracentrifugation (27, 28).
- **Ultrafiltration:** It is another efficient method that relies on the specific size of the pore membrane used to isolate the desired particles and remove contaminants, and during ultracentrifugation EV through the filter collected on precipitation (29). However, albumin in the urine can bind to the surface of EV that might adhere to the nanomembrane and lead to high concentrations of soluble proteins that cannot be removed, limiting the isolation of EV (30).
- **Immunoaffinity capture:** Based on specific surface proteins of EV, monoclonal antibodies with magnetic nanoparticles are used to incubate samples and the conjugate complex captured by magnets. However, the choice of specific markers should be made cautiously because the transmembrane proteins of EV may only recognize a subgroup of exosomes or microvesicles which does not reflect all EV (18, 31).
- Hydrostatic filtration dialysis (HFD): The dialysis membrane with a molecular weight cut-off of 1000KDa is used to isolate EV and hydrostatic pressure of urine pushes the urine through the dialysis membrane to separate urinary EV (32).

However, as a young branch, the urinary EV proteome in DN faces methodological obstacles and lacks an overall understanding. This meta-analysis was intended to review the status quo of urinary EV proteome in DN patients in methodology and proteome aspects. Besides that bioanalysis method was used to unearth the disease mechanism related to urinary EV.

## Methods

### Search strategy

To review the status quo of the studies on urinary EV protein in diabetic nephropathy, we searched the PubMed, Embase, Web of Science, and China National Knowledge Infrastructure (CNKI) databases until January 4, 2022, The search strategy and Participant, Index test, Comparison, Outcome, and Study (PICOS) design strategy are shown in **Table 1**. In addition, the online databases of EV proteins were also retrieved, (http://www.exocarta.org/) open access provided heterogeneous datasets of exosomes and included studies all meeting the minimum experimental requirements of the International Society for Extracellular Vesicles (ISEV)  (6); and EVpedia (http://evpedia.info) a free-web-based database of publications and EV components (33). And all qualified studies were published peer-reviewed. Two reviewers independently screened the abstracts of the retrieved articles; if necessary, the full text was reviewed. Discrepancies were finalized through discussion to reach a consensus.

**Table 1:** The search strategy and Participant, Index test, Comparison, Outcome, and Study (PICOS).

Participant	#1 (diabetic kidney disease[MeSH Terms]) AND (diabetic nephropathy[MeSH Terms])
Index test	#2 ((exosome[MeSH Terms]) AND (microvesicle[MeSH Terms])) AND (extracellular vesicles[MeSH Terms])
Comparison	None
Outcome	#3 (proteome[MeSH Terms]) AND (proteomics[MeSH Terms])
Study design	None
Search	#1 AND #2 AND #3
Language	English and Chinese
Electronic database	PubMed, Embase, Web of Science and China National Knowledge Infrastructure (CNKI)

Selection criteria

The following inclusion criteria were used:

(1): Patients who are diagnosed as DN based on the presence of albuminuria and/or a reduced estimated glomerular filtration rate (eGFR) without signs or symptoms of other primary causes of renal damage, or based on pathological diagnosis (2);: Studies evaluated the urinary EV proteome of DN patients

The exclusion criteria were as follows:

(1) Duplicate articles (2), animal studies or *in vitro* experiments (3), Metabolitics (4), Not urinary exosome (5), Methodology article (6), RNA of urinary exosome (7), Other diseases (8), Comparison effect of pharmaceutical intervention (9), Review or comment.

### Data extraction

We extracted the following information from each eligible study: first author, publication year, study country, categories of cohorts; gender, age and size of groups, a specific type of EV (microvesicles or exosomes) with specific diameter, diagnosis criteria, and methods of urinary EV extraction. In the aspect of protein information, we extracted all reported proteins from eligible studies and the methods of identifying proteins. In addition, we attempted to contact the corresponding authors of the included papers to obtain the original data or the effective size of differential diagnosis in each enrolled study.

### Quality assessment

We appraised the quality and risk of bias of the included studies according to the Newcastle–Ottawa Quality Assessment Scale (NOS). The total NOS score was nine points, including three aspects: selection, comparability, and exposure (34).

### Bioinformatics analysis

All protein and gene names were corrected using UniProt to achieve standardization. Gene ontology (GO) analysis of molecular function was performed using PANTHER GO-Slim 14.0 (http://www.pantherdb.org/) containing the comprehensive, curated database of protein families, trees, subfamilies, and functions (35). All statistically enriched terms, such as GO/KEGG pathways, and hallmark gene sets, based on the default choices under express analysis, as well as cumulative hypergeometric p-values and enrichment factors were calculated and used for filtering. The remaining significant terms were then hierarchically clustered into a tree based on kappa statistical similarities among their gene memberships. The KEGG pathways and protein-protein interaction (PPI) networks were constructed using STRING (https://string-db.org/).

## Results

### Search results and study characteristics

**Figure 2:**
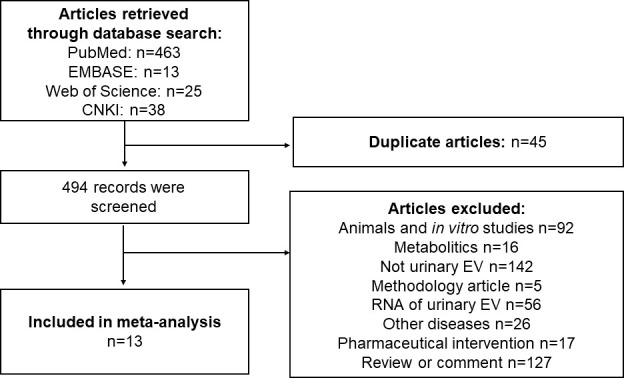
Flow chart of studies selected for systematic review and meta-analysis.

The workflow of the literature selection process is shown in **Figure 2**. In brief, 539 articles were found *via* an initial literature search of the PubMed, Embase, Web of Science, and China National Knowledge Infrastructure (CNKI) databases, and 45 studies were excluded owing to replication. After screening the titles and abstracts, 481 studies were not considered relevant to the purpose of this systematic review and meta-analysis. Subsequently, 13 potential records were subjected to full-text scrutiny, two studies aimed at T1DM (36, 37) two studies enrolled patients with T1DM and T2DM (38, 39), and other studies focused on T2DM patients. 13 studies with 988 participants were enrolled, the characteristics and the diagnosis of each study were shown in **Supplementary Table 1**. Among these, four studies were conducted in China (40–43), three studies were conducted in Spain (38, 39, 44): and two were in Japan (45, 46); besides that, Poland (13), USA (47), India (36), and Ireland (37) performed one related research. DN was defined according to Urinary albumin/creatinine ratio (UACR), urinary albumin excretion rates (UAER) or albuminuria in nine studies; and defined by decreased GFR in one study (13), three studies did not provide description of the definition (38, 39, 44).

### NOS assessment

The quality and risk of bias of the included studies were appraised according to the Newcastle–Ottawa Quality Assessment Scale (NOS). The total NOS score is nine points, including three aspects: selection, comparability, and exposure (34). According to the final score, the studies were categorized into high quality (score > 6), medium quality (4 < score ≤ 6), and low quality (score ≤ 4) (34). The appraisal details are shown in **Table 2**, in which the medium-quality studies were six studies, while seven studies were high quality; the corresponding studies for the medium-quality studies and high-quality studies were enrolled. And all 13 eligible were appraised as reliable for further analysis.

**Table 2:** Quality assessment under Newcastle-Ottawa scale.

Study	Case definition	Representativeness of the cases	Selection of controls	Definition of controls	comparability	Ascertainment of exposure	Same method for ascertainment for both cases and controls	Non-response rate	Score
Benito-Martin, 2013 (44)		*	*	*		*	*		5
Shankhajit De, 2017 (45)		*	*	*		*	*	*	6
Gudehithlu, 2015 (47)	*	*		*	**	*	*	*	8
Kalani, 2013 (36)		*	*	*	**	*	*	*	8
Sakurai, 2019 (46)	*	*	*	*		*	*		6
Sun, 2012 (40)		*	*	*	**	*	*		7
Zubiri, 2015 (38)	*	*	*	*	*	*	*		7
Zuviri, 2014 (39)		*	*	*	*	*	*		6
Kaminska, 2016 (13)		*	*	*	**	*	*		7
Luca Musante, 2015 (37)	*	*			*	*	*		5
Wu, 2018 (41)	*	*	*	*	*	*	*		7
Wang, 2020 (42)	*	*	*	*	*	*	*		7
Chen, 2021 (43)	*	*			*	*	*		5

*: The evaluation indicator had been conducted by the study.

**: In comparability section, if the most impartant factors were controlled then get *, if other second important factors were also controlled then get**

### Isolation and characterization of Urinary EV

In the aspect of the sample, four studies collected the first-morning urine (13, 37, 42, 43) and morning urine with protease inhibitors cocktail were also chosen (38, 39, 44, 45, 47). Spot urine (36) and 24-hour urine (40, 41) were used to isolate urinary EV.

The result of this systematic review showed concordance with preceding studies that ultracentrifugation with various adjusted details was the most widely adopted method to isolate EV. Among the 13 eligible studies, the ultracentrifugation method was operated to isolate urinary EV in three studies (13, 36, 47), and also combined with DTT to eliminate the disulfide bonds of polymerized THP (38, 44). Immoaffiniy depletion kit (38) or 0.22μm filtration (42) were combined with ultracentrifugation to remove high abundance proteins such as IgG or albumin. Besides that, sucrose density gradients (46) or detection kits of markers of urinary EV such as CD63, and CD81 (45) were combined with ultracentrifugation to purify precipitation.

The hydrostatic filtration dialysis method was used in two studies (37, 41), and immunoprecipitation was used to extract urinary EV in one study (40), Exoquick^®^ commercial reagent as well as been chosen in one study (43).

It is worth mentioning that one study comparised three isolation methods: ①ultracentrifugation ②DTT+ ultracentrifugation and ③Exoquick^®^ commercial reagent, and three commercial kits to deplete high abundance protein: ①Albumin Removal SwellGel Discs (Pierce) ②Albusorb^®^ (Biotech Support Group) and ③ProteoPrep^®^ Immunoaffinity Albumin & IgG Depletion Kit (39).

Urinary EV characterization was generally evaluated from three aspects: morphology, size of vesicles and specific markers of EV (8). Eight of 13 studies observed the morphology of urinary EV used TEM technique (13, 38–42, 44, 45). And two studies characterized the size of vesicles using the NTA technique, one of studies identified the exsomes as diameter less than 130 nm, microvesicles as diameter above 130 nm (45), and another of studies the obtained vesicles were 174.6 ± 10.3nm in diameter (41) and more details shown in **Table 3**.

**Table 3:** Methodology of urinary exosome extraction and characterization in eligible studies.

Study	Sample	Urinary EV isolation methods	Characterization		
			TEM	NTA	WB
Benito-Martin, 2013 (44)	Morning urine with protease inhibitors cocktail	Ultracentrifugation + DTT	*		TSG101, CD63
Shankhajit De, 2017 (45)	Fresh second urine with protease inhibitors	Ultracentrifugation + CD63 or CD81 isolation kit (Invitrogen)	*	*	CD81, TSG101, ALIX
Gudehithlu, 2015 (47)	Fresh urine sample (50–100mL) with 2.5 mM DTT and protease inhibitors cocktail	Ultracentrifugation			
Kalani, 2013 (36)	Spot urine	Ultracentrifugation			TSG101
Sakurai, 2019 (46)	Urine samples (50–150 mL)	Sucrose density gradients+ ultracentrifugation			CD63, CD81, CD9
Sun, 2012 (40)	24-hour urine	Immunoprecipitation	*		
Zubiri, 2015 (38)	Urine with protease inhibitors cocktail	DTT+ ultracentrifugation+ depleted albumin and IgG using ProteoPrep Immunoaffinity Albumin and IgG Depletion Kit (Sigma)	*		Alix, calnexin
Zubiri, 2014 (39)	Second-morning urine with a protease-inhibitors cocktail	Comparison of three methods: ①ultracentrifugation ②DTT+ ultracentrifugation ③Exoquick^®^ commercial reagent Comparison of three commercial kits to deplete high abundance protein: ①Albumin Removal SwellGel Discs (Pierce) ②Albusorb^®^ (Biotech Support Group) ③ProteoPrep^®^ Immunoaffinity Albumin & IgG Depletion Kit	*		Alix, TSG101, Calnexin
Kaminska,2016 (13)	First-morning urine (50mL)	Ultracentrifugation	*		
Luca Musante, 2015 (37)	First-morning void urine (15mL)	Hydrostatic filtration dialysis			TSG101
Wu Fan, 2018 (41)	24-hour urine	Hydrostatic filtration dialysis	*	*	TSG101
Wang Lili, 2020 (42)	First-morning urine (100mL)	0.22μm filtration + ultracentrifugation	*		TSG101, CD63
Chen Zhengxu, 2021 (43)	First-morning urine (10mL)	Exoquick^®^ commercial reagent			

*: The method of characterization had been conducted.

### Urinary EV proteins of DN

**Figure 3:**
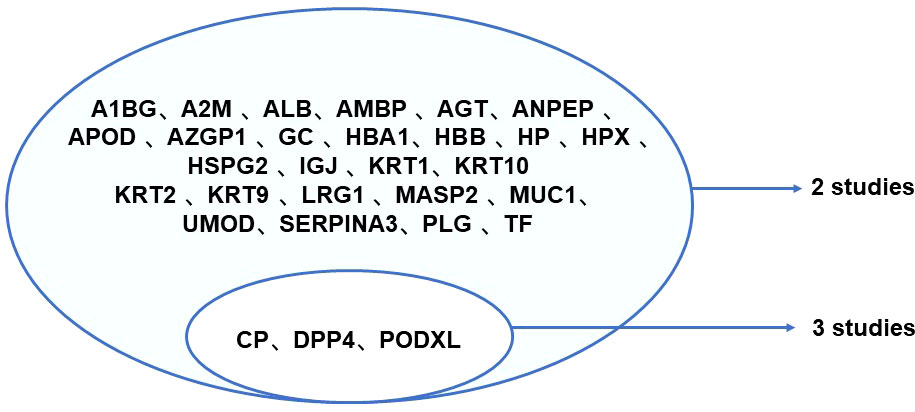
Replicability of urinary EV proteins detected in eligible studies. A1BG, Alpha-1-B Glycoprotein; A2M, Alpha-2-Macroglobulin; ADAM9, a disintegrin and a metalloprotease 9; ALB, Albumin; AMBP, Alpha-1-Microglobulin/Bikunin Precursor; ANPEP, Alanyl Aminopeptidase, Membrane; APOD, Apolipoprotein D; AZGP1, Alpha-2-Glycoprotein 1, Zinc- Binding; CP, ceruloplasmin; DPP4, dipeptidyl peptidase-IV; GC, guanylate cyclase; HBA1, Hemoglobin Subunit Alpha 1; HBB, Hemoglobin Subunit Beta; HP, haptoglobin; HPX, hemopexin; HSPG2, Heparan Sulfate Proteoglycan 2; IGJ, Immunoglobulin J; LRG1, Leucine Rich Alpha-2-Glycoprotein 1; MASP2, mannanbinding lectin serine protease 2; MUC1, mucins; PLG, Plasminogen; PODXL, Podocalyxin; SERPINA3, Serpin Family A Member 3; F, Transferrin; UMOD, Uromodulin.

Among the 13 eligible studies, two researches conducted proteomics studies, that one performed liquid chromatography-mass spectrometry (LC-MS) (39) and another used nano-liquid chromatography coupled offline with mass spectrometry (MALDI-TOF-MS) (13) to obtain the protein profile, and only one study provided the fold change, which could be accessed from the Exocarta database. After scrupulous scrutinize all eligible researches, a total of 579 proteins in urinary EV were retrieved with a minimum cut-off value of two peptides identified per protein, and the list of them were shown in **Supplementary Table 2** with the corresponding references. To evaluate the replicability of studies, all detected urinary EV proteins were summarized and the results shown in **Figure 3**. That 26 proteins were reported in two studies, CP, DPP4 and PODXL were identified in three studies. The results with significant differences among studied groups were shown in **Table 4**, which could become potential biomarkers of DN in the further studies.

**Table 4:** The results of potential biomarkers of DN in 13 eligible studies.

Potential biomarkers	Refence	Results	Method
AMBP	Zubiri, 2014 (39) Kaminska,2016 (13)	AMBP was increased in DN group.	LC–MS/MS SRM Nano-LC-MALDI-TOF/TOF MS
OPG	Benito-Martin, 2013 (44)	OPG in urinary EV was expressed higher in CKD (DN, IgAN and CAKUT) patients, compared with healthy control.	WB ELISA
C-Megalin	Shankhajit De, 2017 (45)	The excretion of C-megalin per urinary EV and C-megalin of total urinary EV were increased with the progression of DN and also positive correlated with UACR. The declined eGFR was negatively correlated with excretion of C-megalin per Urinary EV, while positive correlated with C-megalin of total urinary EV.	WB
MMP MMP2	Gudehithlu, 2015 (47) Luca Musante, 2015 (37)	MMP significantly decreased in DN patients compared with healthy control. MMP2 showed a progressive decrease trend from T1DM patients to DN patients with macroalbuminuria.	ELISA Gel Electrophoresis, WB
CP	Gudehithlu, 2015 (47)	CP in urinary EV significantly increased, compared with healthy control.	ELISA
WT1	Kalani, 2013 (36)	The detection rate of WT1 in urinary EV was significantly higher than diabetic patients than healthy control. The levels of WT1 were significantly higher in DN patients than diabetic patients, and the level of WT1 were associated with a significant increase in UACR and Scr, as well as a decline in eGFR. ROC analysis showed that WT1 effectively predict GFR< 60 ml. min-1/1.73 m^2^.	WB
Elf3	Sakurai, 2019 (46)	Elf3 in urinary EV was only detected in DN patients, and can be a biomarker for podocyte injuries and predict the decline in eGFR in the coming years.	WB
DPP4	Sun, 2012 (40)	The levels of DPP4 in urinary EV were significantly higher in T2DM and DN patients than healthy control group. And macroalbuminuria DN patients were detected higher level of DPP4 than other groups.	ELISA
	Chen, 2021 (43)	The levels of DPP4 in urinary EV: healthy control < T2DM < DN with microalbuminuria <DN with macroalbuminuria.	ELISA
	Luca Musante, 2015 (37)	The T1DM and DN with microalbuminuria patients showed significantly lower level of DPP4 in urinary EV compared with healthy control. Macroalbuminuria patients had higher level and lower functional activity of it compared with healthy control.	Gel Electrophoresis, WB Spectrophotometric Assay
RGN	Zubiri, 2015 (38)	RGN was undetectable in DN patients.	WB
MLL3 VDAC1	Zubiri, 2014 (39)	MLL3 were increased and VDAC1 decreased in DN group. Setting a minimum of 2 assigned peptides per protein, 562 proteins in urinary EV were identified.	LC–MS/MS SRM
CD59 MASP2	Kaminska,2016 (13)	CD59 and MASP2 were found in DN patients.	Nano-LC-MALDI-TOF/TOF MS
CTSA CTSC CTSD CTSE CTSL1 CTSZ KLK10 KLIK13 MME PRTN3 ADAM9	Luca Musante, 2015 (37)	The cathepsin family of A, C, D, L, and Z appeared to progressively increase from T1DM patients to DN patients with macroalbuminuria, while only cathepsin E decreased following trend. PRTN3 has an opposite trend with a marked increase in the normoalbuminuric and microalbuminuria group to reach a normal level in the macroalbuminuric group, and it significantly higher than healthy control. Whereas the level of KLK10 showed same trend with increased albuminuria from T1DM patients to DN patients with macroalbuminuria. MME was detected lower in T1DM and DN patients compared with healthy control. And KLK13in T1DM and DN patients was higher and ADAM9 lower than healthy control.	Gel Electrophoresis WB
PODXL	Wu, 2018 (41)	The level of PODXL in urinary EV showed significantly higher in DN patients, compared with healthy control, T2DM and other glomerular nephropathy patients.	ELISA
IL1B CDH1	Wang, 2020 (42)	The level of IL1B in urinary EV: healthy control < T2DM < DN with microalbuminuria <DN with macroalbuminuria. CDH1 in urinary EV showed markedly lower in T2DM and DN patients compared with healthy control, while it showed no significant differences in T2DM and DN groups.	WB, ELISA

ADAM9, a disintegrin and a metalloprotease 9; AMBP, Alpha-1-Microglobulin/Bikunin Precursor; CAKUT, congenital anomalies of the kidney and urinary tract; CD59, inhibitor of the complement membrane attack complex; CDH1, E-cadherin; CKD: chronic kidney disease; CP, ceruloplasmin; CTSA, cathepsin A; CTSC, cathepsin C; CTSD, cathepsin D; CTSE, cathepsin E; CTSL1, cathepsin L; CTSZ, cathepsin X/Z/P; DN, diabetic nephropathy; DPP4, dipeptidyl peptidase-IV; eGFR, estamited glomerular filtration rate; Elf3, eukaryotic initiation factor 3; EV, extracellular vesicles; HC, healthy control; IgAN, IgA Nephropathy; IL1B, Interleukin-1 beta; KLK10, Kallikrein Related Peptidase 10; KLK13, Kallikrein Related Peptidase 13; MASP2, mannan-binding lectin serine protease 2; MLL3, Myeloid-lineage leukemia protein 3 homolog; MME, Neprilysin; MMP, Gelatinase; MMP2, matrix metallopeptidase 2; OPG, Osteoprotegerin; PODXL, Podocalyxin; PRTN3, proteinase 3; RGN, Regucalcin; ROC, receiver operating characteristic curve; Scr, serum creatinine; UACR, urine albumin-to-creatinine ratio; VDAC1, Voltage Dependent Anion Channel 1; WB, Western blotting; WT1, Wilm’s Tumor-1.

**Figure 4:**
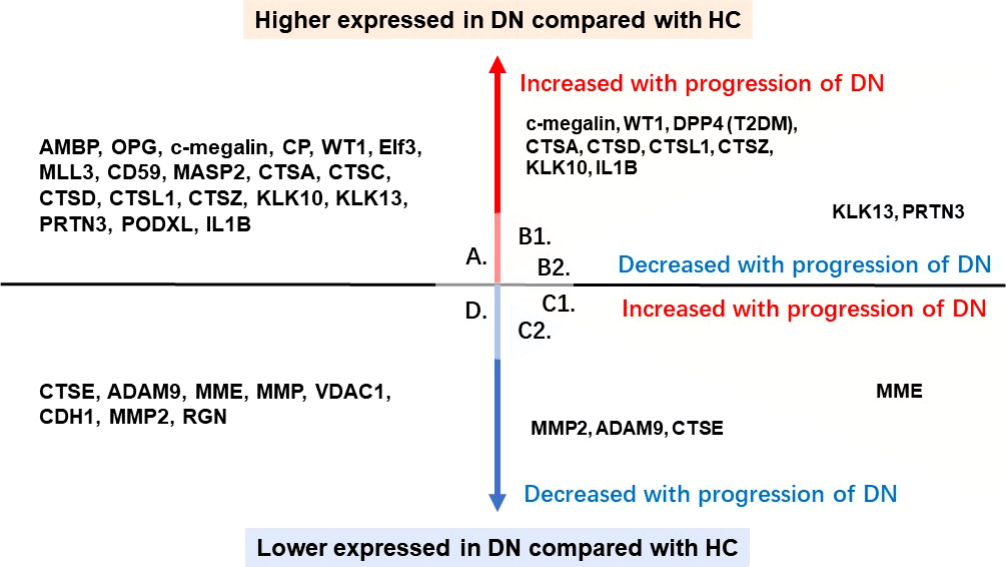
Proteins of urinary EV with significant difference. A. the proteins in urinary EV significantly upregulated in DN patients compared with healthy control; B1. the proteins in urinary EV significantly upregulated in DN patients compared with healthy control and also showed an increased trend with the progression of DN; B2. the proteins in urinary EV significantly upregulated in DN patients compared with healthy control, whereas showed a decreased trend with the progression of DN; C1. the proteins in urinary EV significantly downregulated in DN patients compared with healthy control, whereas showed an increased trend with the progression of DN; C2. the proteins in urinary EV significantly downregulated in DN patients compared with healthy control and also showed a decreased trend with the progression of DN; D. the proteins in urinary EV significantly downregulated in DN patients compared with healthy control.

Compared with healthy control group, there were several proteins in urinary EV significantly upregulated in DN patients that alpha-1-Microglobulin/Bikunin Precursor (AMBP) (13, 39), osteoprotegerin (OPG) (44), c-megalin (45), ceruloplasmin (CP) (47), Wilms’ tumor-1 (WT1) (36), eukaryotic initiation factor 3 (Elf3), isoform 1 of histone-lysine N-methyltransferase MLL3 (MLL3), inhibitor of the complement membrane attack complex (CD59), mannan-binding lectin serine protease 2 (MASP2), and the family members of cathepsin including cathepsin A, C, D, L and cathepsin X/Z/P. Besides that kallikrein 10 (KLK10), kallikrein 13 (KLK13), proteinase 3 (PRTN3), podocalyxin (PODXL) and interleukin-1 beta (IL1B) were shown markedly increased expression in urinary EV than healthy group which were shown in **Figure 4A**.

While cathepsin E (CTSE), a disintegrin and a metalloprotease 9 (ADAM9), neprilysin (MME), gelatinase (MMP), voltage dependent anion channel 1 (VDAC1), E-cadherin (CHD1) decreased in DN patients compared with healthy control, and matrix metallopeptidase 2 (MMP2) showed a progressive decrease trend from T1DM patients to DN patients with macroalbuminuria. And regucalcin (RGN) was undetectable in DN patients. The downregulated proteins in urinary EV were shown in **Figure 4D**.

Besides that, The declined eGFR was negatively correlated with excretion of C-megalin per Urinary EV, while positive correlated with C-megalin of total urinary EV. The level of WT1 were associated with a significant increase in UACR and Scr, as well as a decline in eGFR. ROC analysis shows that WT1 effectively predict GFR< 60 ml. min-1/1.73 m^2^. Elf3 in urinary EV was only detected in DN patients, and can be a biomarker for podocyte injuries and predict the decline in eGFR in the coming years.

The dipeptidyl peptidase-IV (DPP4) in urinary EV was shown significantly differences among studied groups in three studies, and two of them showed same results that the levels of DPP4 in urinary EV were significantly higher in T2DM and DN patients than healthy control group and macroalbuminuria DN patients were detected the most highest level of DPP4 than other groups (40, 43). However, The T1DM and DN with microalbuminuria patients showed significantly lower level of DPP4 in urinary EV compared with healthy control and DN with macroalbuminuria patients had higher level and lower functional activity of it compared with healthy control, which was contradiction with other two studies (37).

### Gene ontology analysis

All detected 579 proteins were analyzed by functional gene ontology (GO) analysis using PANTHER GO-Slim 14.0 to reveal the molecular function, biological process and pathway.

**Figure 5:**
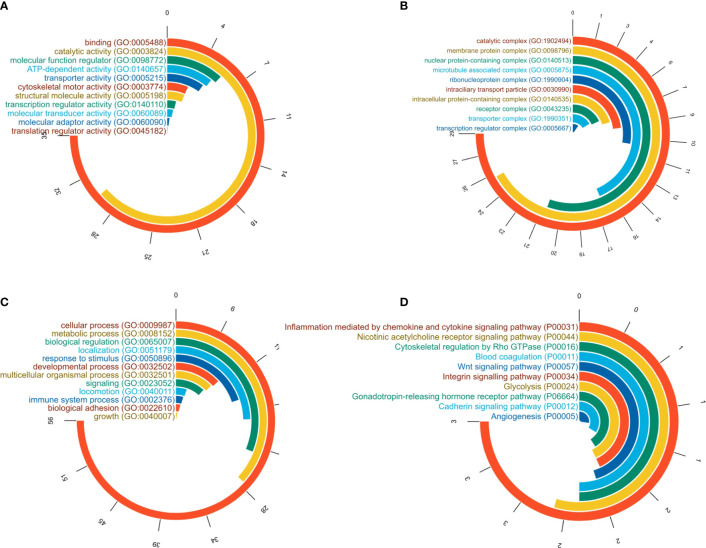
GO analysis of all reported proteins. **(A)** molecular function analysis; **(B)** cellular components analysis; **(C)** biological process analysis; **(D)** pathway analysis.

The analysis of molecular function revealed that the most enriched category is binding (GO:0005488) occupied 35.20% of all proteins. And in the order of hitted number the most enriched molecular functions are as followed, catalytic activity (GO:0003824)accounted for 29.80%, molecular function regulator (GO:0098772) accounted for 5.50%, ATP-dependent activity (GO:0140657) accounted for 5.00%, transporter activity (GO:0005215) accounted for 4.60%, cytoskeletal motor activity (GO:0003774) accounted for 3.00%, structural molecule activity (GO:0005198)accounted for 3.00%, transcription regulator activity (GO:0140110) accounted for 1.90%, molecular transducer activity (GO:0060089) accounted for 1.70%, and molecular adaptor activity (GO:0060090) and translation regulator activity (GO:0045182) were respectively accounted for 1.00% and 0.40% of all urinary EV proteins, which were visualized in **Figure 5A**.

The results of cellular components analysis were shown in **Figure 5B**, that 28.60% of urinary EV proteins were enriched in catalytic complex (GO:1902494), and the second enriched component was membrane protein complex (GO:0098796) accounted for 25.50%, and the following one is nuclear protein-containing complex (GO:0140513) accounted for 21.10%. In the order of hitted number the enriched cellular conponents are as followed, microtubule associated complex (GO:0005875) occupied 16.60% of proteins, ribonucleoprotein complex (GO:1990904)accounted for 10.60%, intraciliary transport particle (GO:0030990) for 8.80%, intracellular protein-containing complex (GO:0140535) for 7.70%, besides that, receptor complex (GO:0043235) and transporter complex (GO:1990351) respectively occupied 6.80% and 6.30% of all reported urinary EV proteins.

Biological process analysis was conducted and the enriched top ten processes were shown in **Figure 5C**. The most enriched was cellular process (GO:0009987) as 56.20% of all reported urinary EV proteins and metabolic process (GO:0008152) occupied with 27.50% as the second enrichment process, biological regulation (GO:0065007) accounted for 23.30%, localization (GO:0051179) hitted number was in four position with 18.50% of all proteins. Besides that, the response to stimulus (GO:0050896) accounted for 14.90%, developmental process (GO:0032502) for 9.80%, multicellular organismal process (GO:0032501) for 9.40%, signaling (GO:0023052) for 8.70%, locomotion (GO:0040011) for 4.00%, and immune system process (GO:0002376) accounted for 3.60% were also noticeable.

In order to further reveal the disease mechanisims, the pathway analysis was also conducted and the results were showen in **Figure 5D**. The most noteworthy pathways were inflammation mediated by chemokine and cytokine signaling pathway (P00031) accounted for 3.30% of all proteins, nicotinic acetylcholine receptor signaling pathway (P00044) accounted for 2.40%, cytoskeletal regulation by Rho GTPase (P00016) accounted for2.20%, blood coagulation (P00011) accounted for 2.20%, Wnt signaling pathway (P00057) accounted for 2.00%, integrin signalling pathway (P00034)accounted for 1.90%, glycolysis (P00024) accounted for 1.90%, and gonadotropin-releasing hormone receptor pathway (P06664) for1.80%, cadherin signaling pathway (P00012) for 1.50% and angiogenesis (P00005) accounted for 1.20%.

A. molecular function analysis; B.cellular components analysis; C. biological process analysis; D.pathway analysis

### PPI network analysis

**Figure 6:**
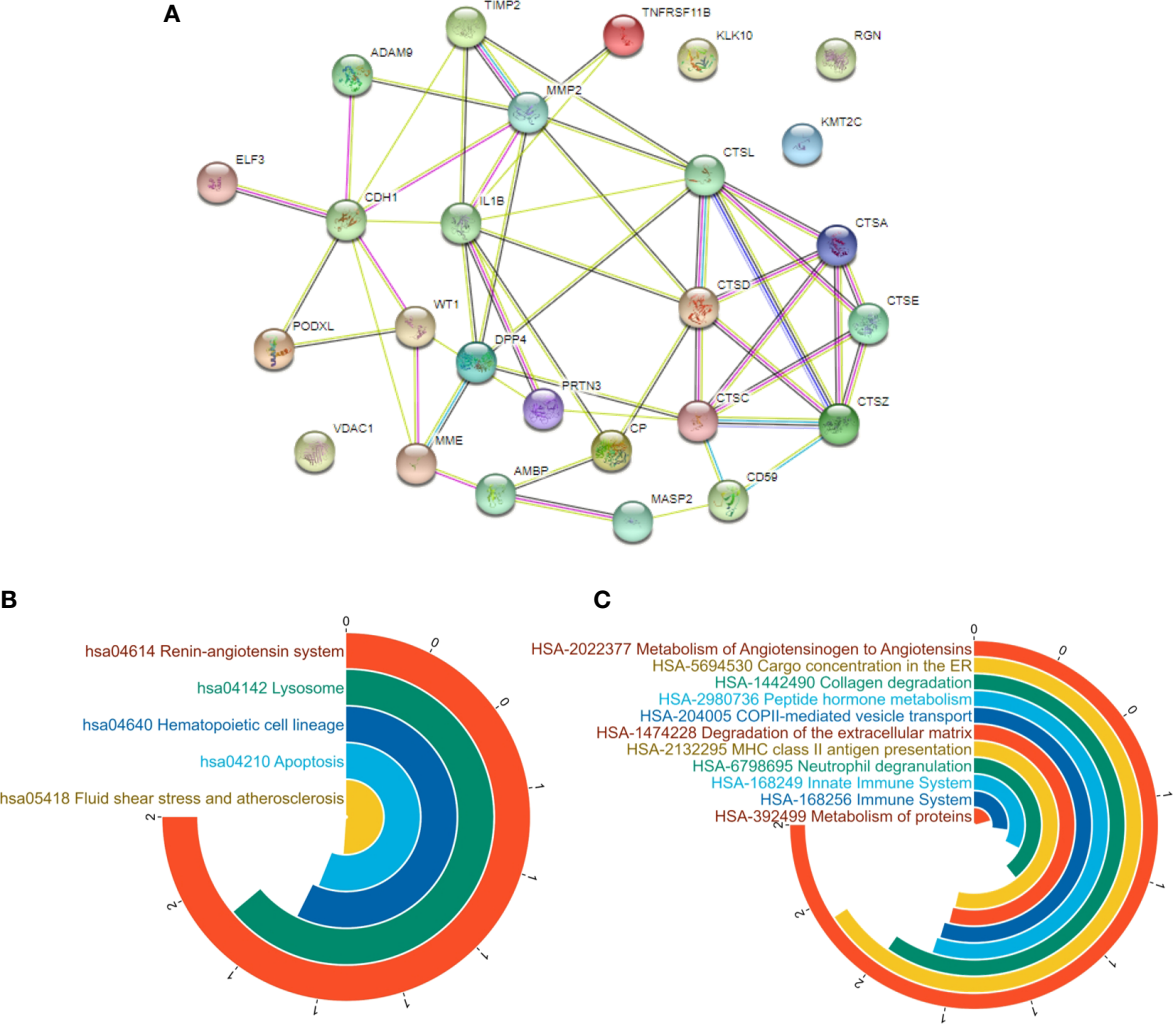
PPI analysis of potential biomarkers. **(A)** the PPI analysis of potential biomarkers; **(B)** KEGG pathways; **(C)** reactcome pathways.

The protein-protein interaction (PPI) network performed to investigate the differential expression and alteration of the protein interaction network of 28 potential biomarkers with the minimum required interaction score set at 0.400. The input protein data were successfully mapped to 26 nodes, with 50 edges and average node degree was 3.85, local clustering coefficient of 0.407, and the results were shown in **Figure 6A**.

Based on the results of KEGG pathways analysis, the top five enriched pathways with the highest strength were hsa04142 lysosome, hsa04210 apoptosis, hsa04640 hematopoietic cell lineage, hsa04614 renin-angiotensin system, and hsa05418 fluid shear stress and atherosclerosis shown in **Figure 6B**. And according to reactcome pathways analysis, the results showed that several pathways had significantly meaning as hsa168256 immune system, hsa1474228degradation of the extracellular matrix, hsa6798695neutrophil degranulation, hsa2132295MHC class II antigen presentation, hsa1442490 collagen degradation, hsa2022377 metabolism of angiotensinogen to angiotensins, hsa2980736 peptide hormone metabolism, hsa-5694530 cargo concentration in the ER, hsa-204005 COPII-mediated vesicle transport, and hsa392499 metabolism of proteins shown in **Figure 6C.**

## Discussion

After summarized the all reported urinary EV proteins, it is notably that DPP4 expressed significantly higher in T2DM and DN patients than healthy control group, and increased with the progression of DN. However, there was a contradictionanry result that in T1DM and DN with microalbuminuria patients showed significantly lower level of DPP4 in urinary EV compared with healthy control, and DN with macroalbuminuria patients had higher level and lower functional activity of it compared with healthy control. This contradiction may be attributed to the difference of between T1DM and T2DM patients, and also could be ascribed to the subgroups of urinary EV leaded by difference of isolation method. That the hydrostatic filtration dialysis, immunoprecipitation, and Exoquick^®^ commercial reagent were respectively used among three studies.

DPP4 is a member of serine protease, widely expressed in proximal tubular in cortex, outer medulla and in the medullary rays, glomeruli, and podocytes which participates in DN *via* increasing GLP/SDF-1, disrupting AGE-RAGE pathway and other various pathways attribute to endothelial to mesenchymal transition (48). In another words, the increased activity of DPP4 is associated with renal injury (37). DPP4-enriched exosomes increased periostin expression in human umbilical vein endothelial cells promoted the angiogenesis (49). Increased angiogenesis is one of key events in diabetes, which was also in accordance with the results of pathways analysis used all reported urinary EV proteins. Therefore, DPP4 in urinary EV increased with progression of DN might act in the angiogenesis which need be further proved (50).

Based on the results of GO analysis conducted in this systematic review that the pathways of lysosome, apoptosis, cargo concentrated in endoplasmic reticulum, and molecular function enriched in binding, cytoskeletal motor activity were all involved with the biogenesis of EV (51). The density of urinary EV of DN patients were siginificantly reduced compared with T1DM (13). However, EV secretion mechanisms remove harmful substance from the plasma membrane, ensuring intracellular environmental balance (52). The substant reduces EV release, and crosstalk between cells about biogenesis and autophagy contributes to maintaining cellular homeostasis under external and internal stresses, leading to chronic kidney disease dysfunction (53). That might reveal the reduced secretion of urinary EV is one of the dangerous signal of progression of DN which could be intervened to improve renal function. In addition, EV contain autophagic cargos that induce autophagy *via* a cascade of target intercellular communication, which plays a pivotal role in the homeostasis of podocytes in DN (11, 54).

The results of bioinformatics analysis also presentated the importance of immune system in the DN. EV regulate the gene expression and signaling pathways of target cells by participating in antigen presentation, transmission of antigenic peptides or immunomodulatory molecules, and regulate adaptive immune response and innate immune response (6). The membrane attack complex C5b-C9 can be removed from the plasma membrane by exosome secretion and excretion mechanisms, ensuring cell survival and recovery from a large number of complement attacks (52). Besides that, the C1q protein binds to the surface of the EV by electrostatic action, and further to the combined with immunoglobulin, triggering the classical pathway of complement cascade activation and triggering the deposition of C3 and C4. Therefore, cells may need to secrete excret more exosomes to compensate for complement activation (55). Phosphatidylserine and histid factors expressed on EV surfaces also activate the coagulation cascade pathway, and thrombin produced during coagulation has C3 and C5 invertase-like activity, further inducing activation of complement cascade diameters (56).

Many studies have detected proteins in urinary EV, however, no studies have identified a specific biomarker to diagnose DN with sensitivity and specificity. whereas WT-1 was a predictor of GFR<60 ml. min−1/1.73 m2 with high sensitivity and specificity. There is still tremendous effort in pathophysiological alterations of urinary EV and DN.

## Conclusion

Based on the PubMed, Embase, Web of Science, and China National Knowledge Infrastructure (CNKI) databases, 13 studies were enrolled into this systematic review and meta-analysis.Two of them enrolled only T1DM patients and two studies enrolled both patients with T1DM and T2DM, and other nine studies focused on T2DM patients. In total 988 participants were enrolled, and DN was basically diagnosed according UACR, UAER or decreased GFR. Totally 579 urinary EV proteins were detected and 28 of them showed potential value to be biomarker in the further studies. The results of bioinformatics analysis revealed that urinary EV may participate in DN through various pathways such as the biogenesis of EV, renin-angiotensin system, fluid shear stress and atherosclerosis, collagen degradation, and immune system. Besides that, it is necessary to report results complianted with the guidline of ISEV, in oder to assure the repeatability and help for further studies.

## Limitation

Few eligible studies conducted MS to obtain the overall map of proteins in urinary EV, and the retrievable data lacked specific figures for comparison among the different studies, which hindered further analysis. In addition, insufficient data prohibit stratified analysis from investigating further the divergence and concordance between different types of diabetes and different severity levels of patients.

## Data availability statement

The original contributions presented in the study are included in the article or **Supplementary Material**. Further inquiries can be directed to the corresponding authors.

## Author contributions

Conceptualization: XD and HZ. Methodology: XD, XW and DZ. Validation: XD, DZ, and JD. Formal analysis: XD. Investigation: XD and XW. Resources: XD and HZ. Data curation: XD and HZ. Writing—original draft preparation: XD. Writing—review and editing: XD, DZ and HZ. Supervision: HZ. Project administration: HZ. Funding acquisition: HZ. All authors contributed to the article and approved the submitted version.

## Funding

This research was funded by The National Natural Science Foundation of China (Nos. 61971441) and the National Key R&D Program of China (2021YFC1005300).

## Acknowledgments

The authors would like to thank Professor Saito Akihiko from the Department of Applied Molecular Medicine, Kidney Research Center, Niigata University Graduate School of Medical and Dental Sciences for providing data search support, and we sincerely appreciate his kindness. **Figure 1** was designed by the first author who used the Figdraw website which had been authorized.

## Conflict of interest

The authors declare that the research was conducted in the absence of any commercial or financial relationships that could be construed as a potential conflict of interest.

## Publisher’s note

All claims expressed in this article are solely those of the authors and do not necessarily represent those of their affiliated organizations, or those of the publisher, the editors and the reviewers. Any product that may be evaluated in this article, or claim that may be made by its manufacturer, is not guaranteed or endorsed by the publisher.
