# Supplementary material for: A systematic review and Meta-analysis of urinary extracellular vesicles proteome in diabetic nephropathy
Source: Front Endocrinol (Lausanne). 2022 Aug 11;13:866252. doi: 10.3389/fendo.2022.866252 (PMC9405893; doi:10.3389/fendo.2022.866252)
Supplement: Supplementary file 1 [file Table_1.docx]

| **Study** | **Country** | **Cohort** | | **Mean age** |
| --- | --- | --- | --- | --- |
| Alberto Benito-Martin 2013(23) | Spain | T2DM with DN n=3 male=3  Glomerular nephropathy n=1 male=1  CAKUT n=1 female=1  ADPKD n=9 male=6 female=3  Healthy Control n=4 male=3 female=1 | | 62±15 |
| Shankhajit De 2017(24) | Japan | Nonalbuminuria-T2DM UACR <30 mg/g n= 20  Microalbuminuria-T2DM UACR 30–300 mg/g n=17  Macroalbuminuria-T2DM UACR ≥300 mg/g n=19  Healthy Control n=19 | | Nonalbuminuria-T2DM: 65.59±12.30  Microalbuminuria-T2DM: 68.65±15.71  Macroalbuminuria-T2DM: 66.63±15.21  Healthy Control: 47.31±9.59 |
| Krishnamurthy P Gudehithlu 2015(26) | USA | Nonalbuminuria-T2DM UACR <30 mg/g n=23 male=10 female=13  Microalbuminuria-T2DM UACR 30–300 mg/g n=23 male=11 female=12  Macroalbuminuria-T2DM UACR＞300 mg/g n=36 10 male=10 female=26  Healthy Control n=20 | | Nonalbuminuria-T2DM 49.9±3.0  Microalbuminuria-T2DM: 52.2±3.1  Macroalbuminuria-T2DM:57.1±2.5 |
| Anuradha Kalani 2013 (14) | India | Nonalbuminuria-T1DM UACR <30 mg/g n=30 female=8 male=22  Albuminuria-T1DM UACR＞300 mg/g n=18 female=4 male=14  Healthy Control n=25 female=6 male=19 | | Nonalbuminuria-T1DM: 33±13  Albuminuria-T1DM: 35±15  Healthy Control: 29±14 |
| Akiko Sakurai 2019(25) | Japan | Macroalbuminuria-T2DM n=50  Healthy Control n=5  DN under renal biopsy n=25 | | 48.2 ± 15.9 |
| Ai-li Sun 2012(19) | China | Microalbuminuria-T2DM UACR 30–300 mg/g n= 50 male=27 female=23  Macroalbuminuria-T2DM UACR＞300 mg/g n= 34 male=20 female=14  T2DM n = 43 male=19 female=24  Healthy Control n=34 male=18 female=16 | | Microalbuminuria-T2DM: 57.12 ± 11.29  Macroalbuminuria-T2DM: 58.47 ± 10.65  T2DM: 57.68 ± 11.28  Healthy Control: 58.97± 10.89 |
| Zubiri, I. 2015(17) | Spain | Healthy Control n=3 male=2 female=1  CKD n=4 male=3 female=1 (T1DM and T2DM) | | 66±12.65 |
| Zubiri, I 2014(18) | Spain | Discovery phase  DN n=5 female=4 male=1 (T1DM and T2DM)  Healthy Control n=5 female=3 male=2 | Confirmation phase  DN n=3 male=3 female=2 (T1DM and T2DM)  Healthy Control n=3 male=2 female=1 | Discovery phase: 63.75±16.21  Confirmation phase: 55.88±5.99 |
| Kamińska, A.J 2016(15) | Poland | T2DM with GFR＜60 mL/min/1.73 m^2^ n=15 female=3 male=12  T2DM with GFR ≥ 60 mL/min/1.73 m^2^ n=45 female=17 male=25  Healthy Control：n=10 female=4 male=6 | | T2DM with GFR＜60 mL/min/1.73 m^2^：69±11  T2DM with GFR≥60 mL/min/1.73 m^2^ 60±3 Healthy Control：52 ± 7 |
| Luca Musante 2015(16) | Ireland | Healthy Control n=12 female=6 male=6  Microalbuminuria-T1DM n=37  T1DM | | Healthy Control：20-40 |
| Wu Fan 2018(20) | China | Nonalbuminuria-T2DM UAER＜30mg/24h n=34 female=17 male=17  Albuminuria-T2DM UAER≥30mg/24h n=23 female=13 male=10  Healthy Control n=11 femal=3 male=8  Other Nephropathy n=21 female=9 male=12 | | T2DM: 48.38±13.31  DN: 55.43±7.28  Healthy Control：43.91±13.70  Other Nephropathy 43.57±13.86 |
| Wang Lili 2020(21) | China | Microalbuminuria-T2DM UACR 30–300 mg/g n= 40  Macroalbuminuria-T2DM UACR＞300 mg/g n = 40  Nonalbuminueia-T2DM UACR＜30mg/g n = 40 | |  |
| Chen Zhengxu 2021(22) | China | Microalbuminuria-T2DM UACR 30–300 mg/g n= 32 female=14 male=11  Macroalbuminuria-T2DM UACR＞300 mg/g n =19 female=9 male=10  Nonalbuminuria-T2DM UACR＜30mg/g n = 46 female=22 male=24  Healthy Control n=31 femal=16 male=15 | | Microalbuminuria-T2DM：56.85±12.27  Macroalbuminuria-T2DM：55.62±11.52  Nonalbuminueia-T2DM：51.69±17.11  Healthy Control：59.56±18.09 |

**Reference:**

14. Kalani A, Mohan A, Godbole MM, Bhatia E, Gupta A, Sharma RK, et al. Wilm's tumor-1 protein levels in urinary exosomes from diabetic patients with or without proteinuria. *PLoS One* (2013) 8(3):e60177. Epub 2013/04/02. doi: 10.1371/journal.pone.0060177. PubMed PMID: 23544132; PubMed Central PMCID: PMCPMC3609819.

15. Kamińska A, Platt M, Kasprzyk J, Kuśnierz-Cabala B, Gala-Błądzińska A, Woźnicka O, et al. Urinary Extracellular Vesicles: Potential Biomarkers of Renal Function in Diabetic Patients. *J Diabetes Res* (2016) 2016:5741518. doi: 10.1155/2016/5741518. PubMed PMID: 28105442.

16. Musante L, Tataruch D, Gu D, Liu X, Forsblom C, Groop PH, et al. Proteases and protease inhibitors of urinary extracellular vesicles in diabetic nephropathy. *J Diabetes Res* (2015) 2015:289734. Epub 2015/04/16. doi: 10.1155/2015/289734. PubMed PMID: 25874235; PubMed Central PMCID: PMCPMC4383158.

17. Zubiri I, Posada-Ayala M, Benito-Martin A, Maroto AS, Martin-Lorenzo M, Cannata-Ortiz P, et al. Kidney tissue proteomics reveals regucalcin downregulation in response to diabetic nephropathy with reflection in urinary exosomes. *Transl Res* (2015) 166(5). doi: 10.1016/j.trsl.2015.05.007. PubMed PMID: 26072307.

18. Zubiri I, Posada-Ayala M, Sanz-Maroto A, Calvo E, Martin-Lorenzo M, Gonzalez-Calero L, et al. Diabetic nephropathy induces changes in the proteome of human urinary exosomes as revealed by label-free comparative analysis. *J Proteomics* (2014) 96. doi: 10.1016/j.jprot.2013.10.037. PubMed PMID: 24211404.

19. Sun AL, Deng JT, Guan GJ, Chen SH, Liu YT, Cheng J, et al. Dipeptidyl peptidase-IV is a potential molecular biomarker in diabetic kidney disease. *Diab Vasc Dis Res* (2012) 9(4):301-8. Epub 2012/03/06. doi: 10.1177/1479164111434318. PubMed PMID: 22388283.

20. Fan W, Yunyin C, Hua X, Ziliang Z, Jing N, Haishan C, et al. Value of podocalyxin levels in urinary extracellular vesicles for diagnosis of diabetic nephropathy (in Chinese). *J South Med Univ* (2018) 38(09):1126-30.

21. Lili W. The expression of IL-1β，TIM-1，E-cadherin in urinary exosomes of diabetic kidney disease (in Chinese) [Thesis of Master Degree ]: Shandong University (2020).

22. Zheng-xu C, Man L, Run-lin Y, Juan J, Bai-yin Z, Yuan-jie Z. Clinical value of the combined detection of urinary exosome DPP-4, Cys C, α1-MG and transferrin in patients with diabetic nephropathy (in Chinese). *J Bengbu Med Coll* (2021) 46(08):1093-5+8. doi: 10.13898/j.cnki.issn.1000-2200.2021.08.027.

23. Benito-Martin A, Ucero AC, Zubiri I, Posada-Ayala M, Fernandez-Fernandez B, Cannata-Ortiz P, et al. Osteoprotegerin in exosome-like vesicles from human cultured tubular cells and urine. *PloS one* (2013) 8(8):e72387. doi: 10.1371/journal.pone.0072387. PubMed PMID: 24058411.

24. De S, Kuwahara S, Hosojima M, Ishikawa T, Kaseda R, Sarkar P, et al. Exocytosis-Mediated Urinary Full-Length Megalin Excretion Is Linked With the Pathogenesis of Diabetic Nephropathy. *Diabetes* (2017) 66(5):1391-404. Epub 2017/03/16. doi: 10.2337/db16-1031. PubMed PMID: 28289043.

25. Sakurai A, Ono H, Ochi A, Matsuura M, Yoshimoto S, Kishi S, et al. Involvement of Elf3 on Smad3 activation-dependent injuries in podocytes and excretion of urinary exosome in diabetic nephropathy. *PloS one* (2019) 14(5):e0216788. doi: 10.1371/journal.pone.0216788. PubMed PMID: 31150422.

26. Gudehithlu KP, Garcia-Gomez I, Vernik J, Brecklin C, Kraus M, Cimbaluk DJ, et al. In Diabetic Kidney Disease Urinary Exosomes Better Represent Kidney Specific Protein Alterations Than Whole Urine. *Am J Nephrol* (2015) 42(6):418-24. doi: 10.1159/000443539. PubMed PMID: 26756605.
